# A Comprehensive Survey on Federated Learning Techniques for Healthcare Informatics

**DOI:** 10.1155/2023/8393990

**Published:** 2023-03-01

**Authors:** K. Dasaradharami Reddy, Thippa Reddy Gadekallu

**Affiliations:** School of Information Technology and Engineering, Vellore Institute of Technology, Vellore, India

## Abstract

Healthcare is predominantly regarded as a crucial consideration in promoting the general physical and mental health and well-being of people around the world. The amount of data generated by healthcare systems is enormous, making it challenging to manage. Many machine learning (ML) approaches were implemented to develop dependable and robust solutions to handle the data. ML cannot fully utilize data due to privacy concerns. This primarily happens in the case of medical data. Due to a lack of precise clinical data, the application of ML for the same is challenging and may not yield desired results. Federated learning (FL), which is a recent development in ML where the computation is offloaded to the source of data, appears to be a promising solution to this problem. In this study, we present a detailed survey of applications of FL for healthcare informatics. We initiate a discussion on the need for FL in the healthcare domain, followed by a review of recent review papers. We focus on the fundamentals of FL and the major motivations behind FL for healthcare applications. We then present the applications of FL along with recent state of the art in several verticals of healthcare. Then, lessons learned, open issues, and challenges that are yet to be solved are also highlighted. This is followed by future directions that give directions to the prospective researchers willing to do their research in this domain.

## 1. Introduction

Healthcare and related services help prevent illness, treat it when it occurs, and promote people's physical well-being. Healthcare providers are increasingly incorporating technology into patient registration, data monitoring, lab testing, and self-care applications. Consequently, people are able to plan ahead while still being in a position to make good decisions about their physical or mental disabilities.

In every organization, data collection is critical. Data can be utilized to forecast current trends and future events. Particularly in healthcare, a large amount of sensitive data is generated and it is very hard to manage and secure the private data. Data security is becoming an increasingly important concern for users. There is a need to develop innovative ways for handling and securing sensitive data in healthcare sectors [1–3]. In order to implement ML models with multiple privacy-preserving methods, it is difficult to construct frameworks and infrastructural facilities [4]. Clinics, moreover, work under stringent privacy standards and can face regulatory, logistical, or ethical restrictions requiring data to stay local. FL is a potential approach for such implementations since it can lighten the stress on a system and allow personal communication among different technologies/institutions [5].

Health records in the public sector are often scattered and confidential, making it difficult to attain reliable outcomes. For example, different clinics have electronic health records (EHRs) with various patient demographics, which are complicated to exchange among hospitals due to their delicate existence [6–10].

FL enables healthcare records that are located across different institutions to be connected without revealing personal information [11].

**Figure 1:**
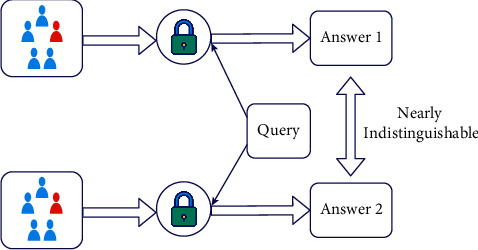
Differential privacy.

Figure 1 shows the result of a differentially private analysis, which ensures that anyone viewing it will conclude the same thing (answers 1 and 2 are virtually the same). The FL concept was first proposed by Google in 2016, for Gboard, a virtual keyboard app for touchscreen mobile devices that supports more than 600 languages. FL effectively overcomes the limitations of classic ML methods by emphasizing data. In contrast to traditional centralized ML methods, which require datasets to reside on a single server, FL minimizes security and privacy concerns by keeping local data stores [12, 13]. FL has distinct privacy advantages over ML models [14, 15]. These special features of FL make it appealing for health research, where a large fraction of the population may want to contribute to novel health findings but have restrictions about sharing their personal and private data.

**Figure 2:**
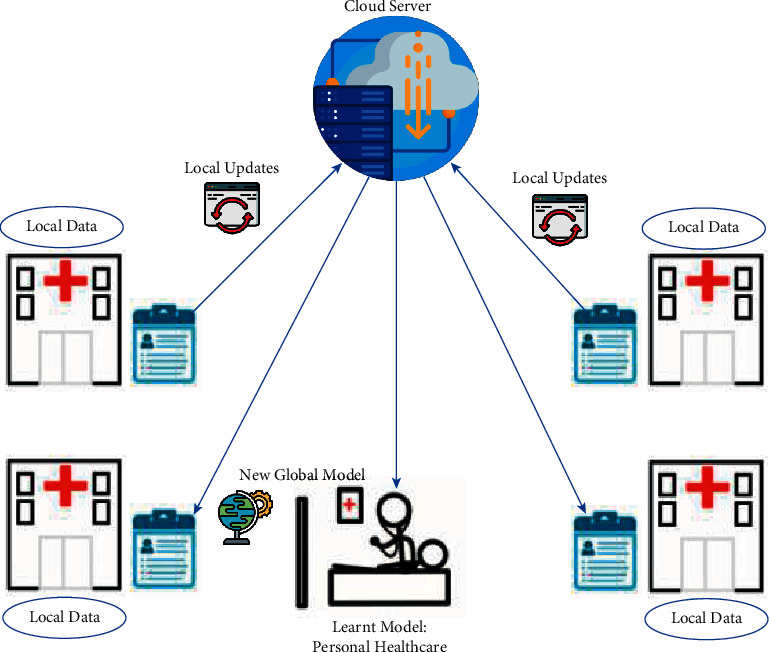
Application of FL for personal healthcare.

Figure 2 illustrates the applications of FL for personal healthcare.

Recently, several researchers have presented survey papers on FL. Dai et al. [16] presented a systematic strategy for replicated statistical analysis in which the Newton-Ralphson technique and an alternating direction method of multiplier (ADMM) framework are employed to conduct shared solutions. Yang et al. [17] describe foundations, infrastructures, and methods for FL, as well as privacy-preserving techniques used for FL. Kumar et al. [18] initiated a unique learning technique that uses blockchain technology to detect COVID-19 in respiratory computed tomography. Xu et al. [19] employed machine learning to diagnose COVID-19 from computed tomography gathered in several clinics across China. These models perform better with FL and also generalize well to models based on just one region. Also, the authors in [20–23] have discussed FL and its applications in various domains.

Similarly, some of the researchers have presented papers on FL for healthcare. Lee et al. [24] gave an example of how to develop patient similarity learning programs that are federated across institutions without protecting the confidentiality of their patients. Kim et al. [25]developed tensor factorization models from massive electronic health records for use in FL environments. Vepakomma et al. [26] designed multiple setups for a global deep learning (DL) algorithm named SplitNN [27] that allows medical groups to train DL algorithms together. Silva et al. [28] studied neural.

Topological correlations across diagnoses, as well as medical samples, illustrate their FL framework. Liu et al. [29] used a distributed strategy to do user presentation modeling as well as overweight-associated phenotyping and got impressive outcomes.

Pfitzner et al. [30] focus on how FL can be applied in healthcare. It implies that real-time data usage is not feasible while preserving patient data. Also, it deals with the pseudonymization of a few fields, which are again retraceable. It discusses how to distribute the load of the training process onto the FL. It identifies some open challenges, like privacy-preserving hyperparameter optimization, entity resolution for vertically split data, and efficient ways of using encryption.

Rieke et al. [31] discuss the current FL efforts for digital health and their impact on stakeholders, clinicians, patients, hospitals and practices, researchers and Artificial Intelligence (AI) developers, healthcare providers, and manufacturers. It highlights FedAvg and FedProx algorithms. It also specifies that though FL has been a challenging solution in neatly addressing issues related to sensitive medical data, which may open novel research and business avenues and can improve patient care globally, there are also so many open technical questions that have not been answered yet.

Xu et al. [11] focus on the current state of FL, including but not limited to the healthcare sector. It discusses the use of FL in healthcare as well as some of the challenges associated with the combination of FL and healthcare, such as incorporating expert knowledge, personalizing health, and obtaining model precision.

Nguyen et al. [32] discuss advanced FL designs that would be useful for federated smart healthcare, as well as the important applications of FL in smart healthcare, such as federated EHRs management, federated remote health monitoring, federated medical imaging, and federated COVID-19 detection.

Antunes et al. [33] examined the systematic literature review of current research about FL in the context of EHR data for healthcare applications. The assessment of these articles reveals a variety of efforts to attain and provide best practices for protecting training data privacy.

A summary of the key findings from the above discussion can be found in Table 1.

The potential of FL in healthcare, such as efficient handling of sensitive medical data, improving the data quality and model precision, and proper electronic health records handling has not been focused on in the open literature [11, 30–32]. Moreover, a comprehensive discussion of the impact of FL on disease diagnosis and applications in medical imaging, the Internet of things (IoT), and the COVID outbreak is still missing. Particularly, we provide a state-of-the-art survey of the applications of FL in various key healthcare services such as medical imaging, IoT, COVID outbreak, managing electronic health records (EHRs), and health cooperation. In the last part of this review, there are some potential directions for FL in healthcare.

For the purpose of better comprehension, the definitions of the abbreviations used in the paper are summarized in Table 2.

## 2. State of the Arts and Contributions

### 2.1. ML for Healthcare

AI methods are increasingly used to support experts in the medical field due to their effectiveness in detecting and classifying diseases. A few ML approaches like prediction, categorization, grouping, and learning techniques are used in various fields, like image analysis, speech recognition, and healthcare [34, 35]. The state of the art of ML techniques used for computer-aided diagnosis to detect breast cancer from various imaging techniques has been investigated [36].

Multiple sectors were being suggested to utilize COVID-19 ML approaches, including clinical practice, healthcare quality forecasting, and monitoring and identifying diseases [37–41]. The use of ML and DL has become a fundamental method of knowledge discovery in several industries. Large, diverse datasets are essential for the success of data-driven applications. However, it is difficult to get medical datasets. FL solves these problems by allowing group work by centralizing information, and it is already being utilized in digital applications across the health field [31].

### 2.2. FL for Healthcare

FL brings together all the healthcare institutions, allowing them to share experiences and ensuring their privacy. In this scenario, the enormous medical dataset will help improve the quality of the ML model. In healthcare, FL is often used to study a range of tasks, like client identity, client knowledge, diagnostics, identifying possible hospitalizations, detecting death rates, intensive care unit admission periods, etc. ML algorithms require vast, comprehensive datasets, and FL provides controlled, indirect access while protecting patient privacy. FL's promise is simple: by permitting ML from pseudo data, it will overcome personal information quality issues. FL environments apply their respective policy implementations with security measures to each provider, and they also monitor Internet connectivity.

**Figure 3:**
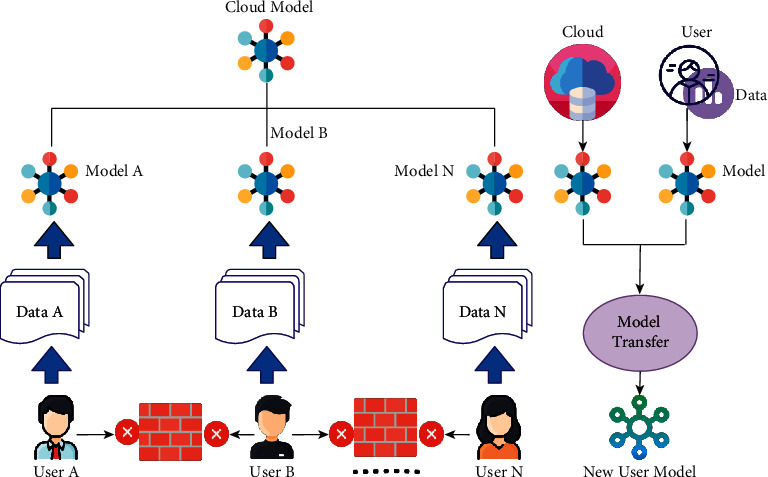
FL for the healthcare system.

Figure 3 shows how FL may affect the way AI models are taught while also benefiting the overall wider healthcare ecosystem.

FL offers great assurances on analytics of health data. There are uses for both providers (such as developing a prediction strategy for such risk of chronic illnesses using electronic health records (EHR) [42]) and consumers (patients) (for example, cardiac medical exam by wearable device with electrocardiograms [43]). The federated model learning process is designed to allow academic researchers to easily maintain sensitive patient data.

Federated Transfer Learning (FTL) is another architecture for FL. FTL [44] is used to train models using data from a different source. FTL recently attracted significant attention in different industries, particularly healthcare [45]. FTL employs cryptography and estimation to verify that confidentiality is successfully preserved to prevent potentially exposed client data. FL is an effective solution for healthcare, as it connects data from multiple silos without requiring a transfer of all patient data. The future of healthcare is dependent on the development of innovative technologies that respect data privacy.

### 2.3. Impact of FL on Disease Diagnosis

The process of developing an understanding of a situation by using clinical reasoning and utilizing information acquired based on observations is called disease diagnosis. There are many steps involved in diagnosing a disease because this is an essential part of medical science. A source of uncertainty exists at every step of the diagnostic process. A diagnosis begins with acquiring knowledge first from the client's diagnostic test, as well as knowledge provided from laboratory tests and other medical diagnosis methods.

A diagnosis has an enormous impact on patients, in terms of both care and research. There have been multiple views on the nature of diagnoses, such as a process and classification scheme, or a previously planned set of categories. Clinical decision-making takes place according to the proper understanding of the health problem of the patient; therefore, accurate and timely diagnosis is key to receiving the best possible health outcome.

The FL method for early diagnosis of Type-2 diabetes uses feature selection algorithms and federated multilayer perceptron models. Furthermore, a comparison between a centralized ML model and a decentralized FL model is made to demonstrate the importance of a decentralized model when it comes to privacy considerations [46].

### 2.4. Paper Organization

The majority of this article is based on the following: Section 2 describes FL's background and motivations.  Section 3 describes applications of FL in verticals of healthcare. Sections 4 and 5 include recommendations regarding future directions of research based on lessons learned from previous reviews.  Section 6 discusses the paper's conclusion.

## 3. Background and Motivations

This section introduces an overview and status of disease diagnosis using FL, fundamentals of FL, and motivations of using FL for healthcare and discussed the main contributions of the survey.

### 3.1. Overview and Status of Disease Diagnosis Using FL

FL is a paradigm that aims to collaborate on data management and privacy issues by using evaluation metrics without transferring samples [47–49]. Initially, this methodology was introduced in a separate discipline, and it has lately been adopted in the medical industry since it overcomes the challenges which are typically encountered while intending to collect patient data. In the context of electronic medicine, it suggests FL allows findings to be obtained collectively between entities without sharing patient data, such as in the form of a universal model. The strength of FL substitutes to prevent sensitive training data movement beyond its firewalls.

Healthcare has been transformed by wearable devices in numerous ways, including improvements in patient care, rehabilitation, and disease management. These devices generate data that can be used to detect early signs of cognitive illnesses like diabetes and dementia. However, the personalization and privacy issues they generate have raised concerns among users. An FL framework called FedHealth is being developed by Chinese researchers in order to minimize the risk of security breaches. FedHealth aims to address the vulnerabilities of today's healthcare industry. FedHealth software creates capable ML models based on FL and homomorphic encryption to protect users' privacy.

**Figure 4:**
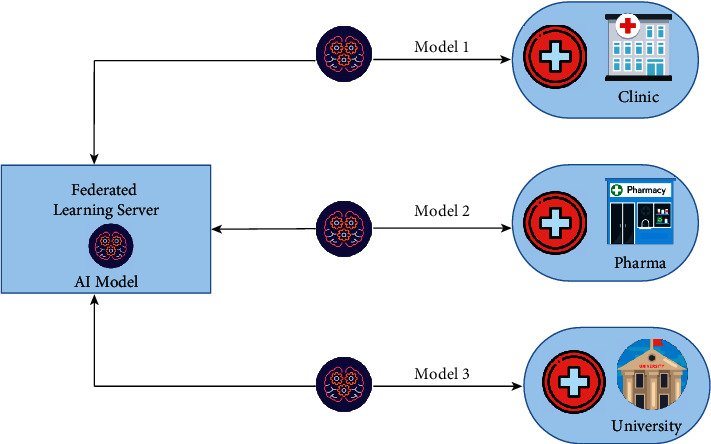
The FedHealth framework in which “user” represents an organization.

It is based on four primary components, according to Jindong. In the first place, the server's cloud model is trained based on publicly available data. It is then disseminated to all users, who may subsequently train the model using their own data. Once you have achieved this, a new cloud model can be created through model aggregation. FedHealth uses transfer learning techniques to ensure that each organization has a uniquely tailored model after improving its cloud model.  Figure 4 depicts the team's framework.

FL allows data isolation to be solved by combining user data to build ML models. FedHealth can update the cloud model and user model simultaneously as soon as new data is available. In this way, the more a wearable device or application is used, the more customized it becomes and how the model is viewed. FedHealth is not currently being implemented, but it does offer new avenues for wearable medical applications. According to Jindong, this framework can perform diagnoses of other diseases and illnesses, including coronavirus disease, using radiology test images.

### 3.2. Fundamentals of FL

Google first suggested federated machine learning [Kone Diecn'y et al., 2016], in which it trained ML models on distributed mobile phones. The main idea during the process is to protect user information. FL is capable of resolving data isolation issues through network model training in the context of privacy preservation. FL makes it possible for several workers to learn a strong and generic ML model by providing information so that key issues like user privacy, information security, authentication rights, and exposure to large datasets are addressed. These technologies cover a variety of sectors, like military, mobile communications, IoT, and healthcare [50].

FL is designed to build a teaching practice with many individual variables containing network elements by actively transferring raw data, for future reference, into deeper neural networks. The main principle consists of training individual designs on available information and generating variables (for example, weights and distortions of a classifier) for the generation of a modeling framework maintained across all connections between neighboring controllers.

**Figure 5:**
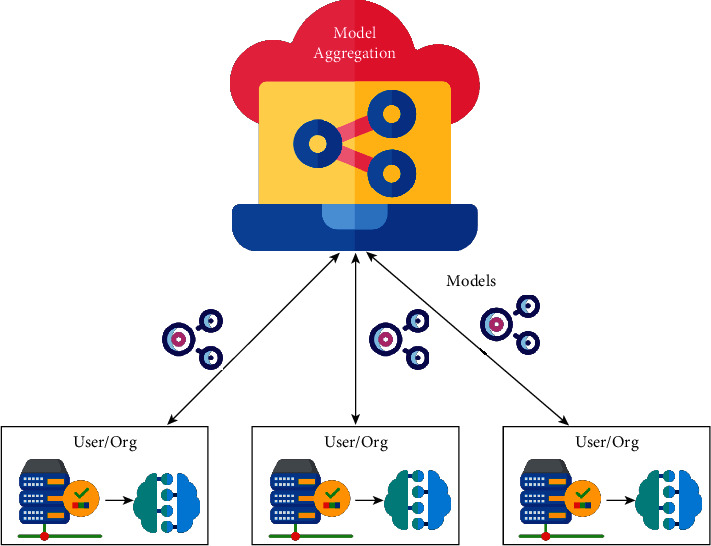
FL architecture.

Figure 5 shows how local model parameters are transferred to a primary controller to generate a large reference model. Once the federated model has been created, it is then returned to the clients for initial training and iterative improvement. Participants can also have their own computing resources. FL will be used in practice by large organizations, and it will be important in promoting security systems whose training data is being spread around the world.

### 3.3. Motivations of Using FL for Healthcare

In healthcare organizations, the primary motivation for using FL is as follows:

- FL reduces data security and privacy concerns by maintaining local data stores, as opposed to centralized ML techniques, which require datasets to reside on one server [51].
- Through FL, large datasets from multiple hospitals can be readily accessed by an individual hospital without centralizing the data into one place. As a result of this practice, critical issues such as data access rights and access to heterogeneous data are addressed.
- The construction of good models without biases using small datasets is very difficult as it takes a lot of time, effort, and cost to collect, curate, and maintain high-quality data from a diverse population. The experts in healthcare and life sciences can use FL to solve the unique problem of data governance by training algorithms behind the hospital's firewall and only sharing models so that data remain secure [31].
- FL captures a wide range of data variables and analyzes patients based on any of their demographic characteristics. For example, with access to electronic health records, FL can help to find clinically similar patients and predict hospitalizations due to cardiac events, mortality and ICU stay time [52].
- FL can have an enormous influence on a variety of stakeholders like clinicians, patients, hospitals, medical researchers, and healthcare providers.
- FL is a potential concept for safe, reliable, and impartial models of data. FL makes it possible for several parties to work together without exchanging or centralizing datasets.
- FL provides AI developers with access to bigger and diversified data packages, which better portray current patients. As a result, AI-based healthcare solutions will be able to scale globally at an unprecedented level.

### 3.4. Main Contributions of the Survey

There are numerous existing works that discuss the enabling technologies, protocols, applications, and challenges of FL. FL facilitates communication between various parties without the need to communicate or centralize data, thereby resolving issues with critical health information. In addition, this could lead to new business opportunities and improved healthcare worldwide. The contributions of our work can be summarized as follows:

- Several terms and definitions for FL for healthcare are gathered from the available literature, resulting in a more comprehensive understanding of FL for healthcare from various perspectives.
- FL is next developing and enabling applications in healthcare such as medical image processing, IoT-based smart healthcare applications, and outbreak prediction.
- Despite several research and development activities, many challenges and issues are imposed in FL for healthcare. We present these difficulties in terms of confidentiality and safety, data heterogeneity, traceability and accountability, and system architecture. We also highlight promising research directions toward the realization of FL for healthcare.
- Finally, we discuss future directions of FL for healthcare applications like healthcare 5.0, including digital twins (DT) in healthcare, FL and blockchain for healthcare, collaborative robots in healthcare, FL and Explainable AI for healthcare, and FL for integration with 6G in healthcare.

## 4. Applications of FL in Verticals of Healthcare

This section discusses some of the potential applications of FL for healthcare.

### 4.1. FL Applications for Medical Image Processing

Medical imaging has transformed the healthcare sector, enabling practitioners and scientists to discover more about the human body than ever before. Medical image processing provides techniques for enhancing and analyzing raw medical image data for selective visualization and analysis according to a given problem. Medical imaging can also help with the treatment and long-term management of a problem. As medical technology advances, doctors can detect problems that are more difficult to detect through simple external examinations. Medical imaging is important for determining the severity of an ongoing illness. Medical imaging refers to a variety of technologies that are used to see the human body in order to diagnose, monitor, or treat medical disorders. Each technology provides unique information regarding the part of the body being examined or treated, whether it is related to disease, injury, or the effectiveness of medical treatment.

Fast and accurate reconstruction of magnetic resonance images (MRIs) from training data is essential in many clinical applications. In recent years, DL-based techniques have been discovered to perform better in MRI reconstruction. However, these methods necessarily involve massive amounts of data, which are difficult to generate and distribute due to high acquisition costs and medical data privacy regulations. To address this issue, we offer an FL-based system that makes use of MRI data from several institutions while protecting patients' privacy [53].

**Figure 6:**
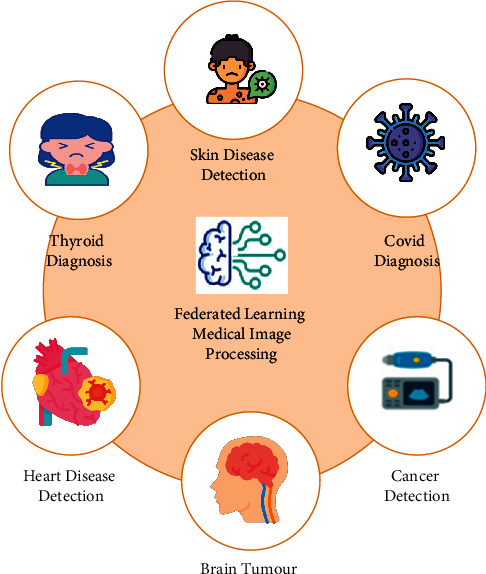
Applications of FL in medical image processing.

The FL algorithm is used to describe and identify physician-related persons and to estimate their hospitalization, deaths, and survival rates based on electronic health records (EHR) [54]. A complete brain part of an MRI [55] is helpful, and for brain tumor segmentation [56], the usage and advantages of FL were further demonstrated. Recently, the fMRI [57] classification technique was used in finding a suitable risk factor for several diseases. FL will help in detecting various diseases as shown in Figure 6.

FL is proposed as a promising approach to COVID-19 detection. Qayyum et al. [58] implement the surging concept of clustered federated learning (CFL) for an automatic diagnosis of COVID-19 using X-ray and Ultrasound datasets.

FL allowed medical professionals to assess the intensity of skin disease. Hashmani et al. [59] proposed an architecture that can diagnose the type of skin disease by conducting experiments using dermoscopy images to test and validate the model's classification accuracy and adaptability.

FL is more potential in analyzing the medical images and also in protecting the patient's privacy. Lee et al. [60] used ultrasound image analysis and FL to determine whether thyroid nodules were normal or dangerous.

FL enables efficient and accurate heart disease diagnosis. Linardos et al. [61] present the first FL study for the diagnosis of hypertrophic cardiomyopathy (HCM) using the subsets derived from *M* and *M* and ACDC datasets.

FL is a privacy-preserving AI model to identify brain tumors. Li et al. [56] focus on practical FL systems for brain tumor segmentation by using the BraTS dataset.

A summary of the key findings from the above discussion can be found in Table 3.

### 4.2. FL in IoT-Based Smart Healthcare Applications

**Figure 7:**
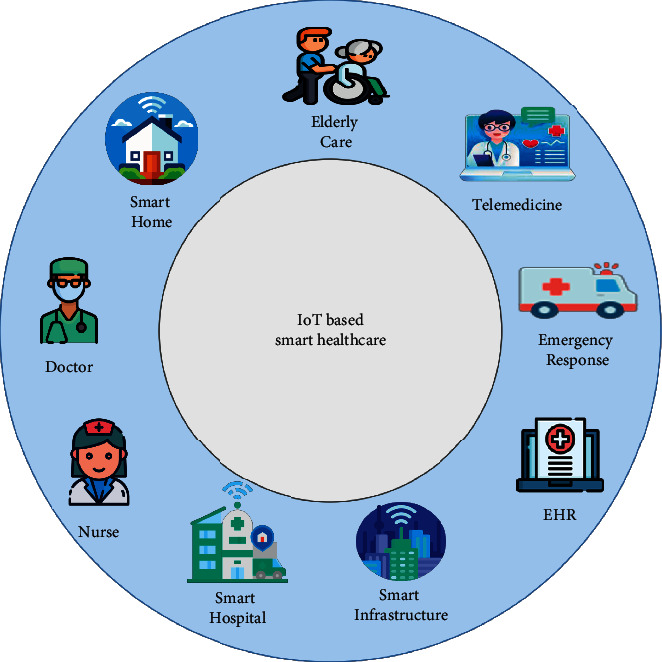
IoT-based smart healthcare.

FL is a concept developed to construct smart and confidential IoT devices. FL has developed a system that makes it possible for doctors to use data from multiple medical institutions without sharing patient data across them [62]. Hospitals can train their own AI models by uploading the data to a global aggregator. FL brings together multiple hospitals to develop collaborative healthcare settings that speed up diagnostic testing of victims while maintaining their personal rights. The healthcare sector is one of the most attractive applications of IoT, as depicted in Figure 7.

The use of AI-based approaches to learn health data has been adopted widely in smart healthcare, such as the use of intelligent imaging to detect disease [63]. Medical information requires a degree of insensitivity and is regulated by medical rules like the Health Insurance Portability and Accountability Act (HIPAA) [64]. Ensuring data protection from public sources shared with its server or storage system is an issue [65]. Traditional AI systems relying on some kind of master database software are not really suitable for contemporary health.

FL's utility in the smart healthcare sector with sophisticated features has been proved in recent research. The role of electronic health records in the healthcare industry is growing rapidly. Here, we focus on two business scenarios, namely, EHR maintenance and involvement in health coverage.

#### 4.2.1. Managing Electronic Health Records Using FL

The application of FL in healthcare operations has really been studied in order to provide adaptable as well as confidential EHR maintenance. An FL-based student-centered architecture is responsible for an EHR system with different clinics as well as a database server [66]. Every company uses its own electronic health records and a cloud server to manage a neural network (NN). A new method of data destruction has been developed by Google. The concept is intended to protect the learning experience in data storage from cyberattacks. It uses a lightweight data destruction technique to disrupt training data and to ensure confidentiality of the FL model parameters that can protect them. Although an attacker can get troubled EHR information, the source data is difficult to gather or retrieve. The AlexNet NN simulation was performed with the CIFAR-10 standard dataset to achieve accurate and safe results for the study of EHR.

Liu et al. [67] built a distributed NN activity plan which enables any clinic to know and understand a part of the model from its EHR source. The integration of autonomous position monitoring and conventional nonconvex enhancement principles [68] builds a different innovative method for FL. The aim is to save network resources by interacting with the remote EHR server. FL allows the sharing of EHRs without their sharing due to its dynamic approach to education. New features to the model suffer anonymity, which leads to communication path inference attacks. To tackle this problem, differential privacy strategies can help enhance the protection of personal data for the learning of FL-based EHRs [69].

EHR information is used for detecting FL's adverse drug reactions (ADRs). It can be used to detect rare ADRs at a single site for rare ADRs and to predict more severe ADRs in the long term [70]. FL delivers similar accuracy in predicting ADR without sacrificing data confidentiality in comparison with centralized AI approaches. The authors of [71] propose that unnecessary changes be deleted by examining the importance of small variations in the FL structure for every EHR user, to increase the quality and precision of integration. FL-based healthcare imaging architectures protect patient privacy as a key feature [72]. In this context, clinics and healthcare physicians cooperate together over the development of a safe and multiparty FL system for service users with medical algorithms.

#### 4.2.2. Health Cooperation FL

In order to contribute, FL can ensure proper healthcare coordination for better provision of medical facilities through its centralized and secure nature. Yuan et al. [73] present a cooperative framework for healthcare that leverages FL to enable medical IoT devices.

The next generation of FL solution is presented in [74] cloud edge-based healthcare. This solution could be used to address issues that directly affect the FL process, such as device, data, and model. Personalization training on edge computers was decided to be carried out, in this case, to reduce heterogeneity and achieve high-quality individual models. For the purpose of stimulating the federation of portable devices using FL technology, the FedHealth framework [75] was developed. Information collected by a number of healthcare institutions using different IoT devices may be collected using Fed- Health's surgical instruments to enable the development of a powerful AI method that can be used to identify people's behavior and to protect ciphertext data [76]. An innovative method [77] is the use of chain-directed synchronous stochastic gradient descent to minimize human latency between FL clients and servers in personal mobile sensing applications. Recent studies reveal that FL is helpful in preventing infections like COVID-19 [78].

A summary of the key findings from the above discussion can be found in Table 4.

#### 4.2.3. FL for COVID Outbreak Prediction

FL utilizes multiple devices or servers that store local samples of data, without aggregating clinical data to create a statistical model, which is undesirable for several reasons, including patient privacy concerns. COVID-19 infection has been confirmed in China since December 2019. After that, the outbreak began spreading to China and several other countries across the globe [79]. A pandemic with rapid growth (and thousands of infections and hundreds of deaths) presents considerable obstacles to control the virus.

There are currently a number of different ways to detect COVID-19, whereas computed tomography and X-rays remain the major surgical techniques [80–82]. A number of clinical symptoms are observed in COVID- 19 patients hospitalized with Acute Kidney Injury (AKI) [83–86]. According to studies, the incidence of AKI ranges between 46 percent and 71 percent; however, most deaths occur within the AKI sample [87, 88]. Diagnostic methods that judiciously help individuals at greater risk of contracting COVID-19 can be beneficial during an outbreak where facilities can be restricted [89].

Despite FL's promise, it is still a relatively new idea for physicians, patients, payers, researchers, and hospitals. FL is strongly encouraged to examine whether and how it can provide valuable support during and after the unprecedented COVID-19 pandemic to control it.

A summary of the key findings from the above discussion can be found in Table 5.

In COVID-19, FL demonstrates its value in diagnosis, treatment, and prognosis prediction. Xu et al. [19] demonstrated that with the help of chest computed tomography (CT) scan data from several institutions in Wuhan, China, they were able to overcome data scarcity, isolation, and heterogeneity and achieve improved detection sensitivity. Based on their findings, an FL-based architecture would allow global participants to benefit from a globally distributed and real-time CT-COVID-19 diagnostic tool. A study by Vaid et al. [92] published recently found that FL-based models tended to perform better than locally trained predictive models when using data from medical centers to predict 7-day mortality in hospitalized patients. The intent of this study is to evaluate FL in predicting a meaningful outcome for hospitalized COVID-19 patients.

A confirmatory finding of FL's superiority is likely to spark significant interest, especially in its potential to improve outcomes for COVID-19 patients. FL has been able to harness all of the full learning power of existing data to offer data-driven insights and personalized recommendations due to the uncertainty of long-term complications of COVID-19, the effectiveness of medical treatments, the safety of vaccines, and immunity protection.

COVID-19 care is only a small part of FL's value as a health provider. This pandemic has severely disrupted non-COVID-19 multicenter clinical trials. It has become increasingly challenging to utilize data generated from each participating institution. In recent years, it has been said that decentralizing clinical research would benefit traditionally underrepresented subgroups and underserved areas in particular. This kind of decentralized research could benefit greatly from integrating FL into the study design and data analysis to evaluate the quality of care and outcomes, for example, predicting mortality, complications, hospitalizations, and adverse drug reactions. Another area for FL to explore is digital health. Healthcare, precision medicine, wearable technology, and clinical decision support have all seen a rise in popularity as a result of the epidemic. The suitable use of FL produces generalizable models that will help the achievement of equitable, effective, and patient-centered care.

FL can effectively address the issue of data unavailability and get a shared model without obtaining local data. Boyi Liu et al. [90] proposed an experiment in which four popular federated ML models (Mobile Net, ResNet18, MobileNet-v2, and COVID-Net) were applied to CXR images of patient's chests to compare their performance. The authors developed these models to detect COVID-19 pneumonia, using the same parameters for all models.

FL proved to be more efficient compared to traditional ML models in COVID detection. Pang et al. [91] created a federated model based on a digital city twin concept to predict outcomes of different COVID-19 prevention plans over time and assess the survival rates of multiple cities over the study period. Furthermore, using the digital city twin platform, they were able to track the effectiveness of each prevention plan and create local models which were sent to the federated sites for safety.

## 5. Lessons Learned

In this section, we summarize the key lessons learned from this survey, which thus provide an overall picture of the current research of FL applications for medical image processing, FL toward privacy and security in healthcare applications, FL in IoT-based smart healthcare applications, and FL for outbreak prediction.

### 5.1. FL Applications for Medical Image Processing

Based on the observations from several states of the art, the FL algorithm analyzes electronic health records to describe and estimate hospitalization, survival, and death rates for people associated with physicians. In addition to identifying disease risk factors using the fMRI classification technique, a promising COVID-19 approach was presented as well.

### 5.2. FL in IoT-Based Smart Healthcare Applications

FL has developed as a distributed collaborative AI approach that has the ability to enable a wide range of intelligent IoT applications by allowing AI training at distributed IoT devices without the need for data exchange [93]. Based on the observations from several states of the art, FL developed a system that allows doctors to access patient data across multiple hospitals without constantly transferring patient information. FL provides smart healthcare solutions and reshapes current healthcare systems by improving privacy for users and reducing latency among healthcare providers and users alike. A lesson to be learned from this is that the FL can enable healthcare operations to manage EHRs centrally and with privacy preserved, by collaborating with multiple medical institutions to build intelligent EHR systems. Further, few fully decentralized FL approaches are able to provide decentralized optimization and stochastic gradient tracking by combining the cooperation of hospitals with a decentralized stochastic gradient algorithm to improve convergence rates.

### 5.3. FL for COVID Outbreak Prediction

Based on the observations from several states of the art, in an outbreak when facilities may be restricted, it is important to develop diagnostic methods that will help individuals at greater risk of contracting COVID-19 judiciously. We also find that using FL in decentralized research would benefit researchers by improving study design and analyzing data on outcomes and quality of care, including predicting mortality, complications, and adverse drug reactions.

Goldfarb et al. [89] consolidate existing case studies and identify the future challenges in defining FL's benefits and impact on healthcare applications, as well as the obstructions and concerns surrounding its adoption in healthcare.

## 6. Challenges and Future Directions

FL does have some advantages, but it also poses challenges that must be considered when setting up federated training efforts.

### 6.1. Confidentiality and Safety

There is a lot of important data in the healthcare industry which needs to be defended. FL poses many privacy-preserving challenges in terms of alternatives, techniques, and consequences.

#### 6.1.1. Privacy versus Performance

FL is primarily designed to safeguard confidentiality by discussing method updates instead of information, although it does not solve many security issues but, like any ML algorithm, always carries some risk. FL offers a level of privacy preservation that is superior to the current commercially available ML models in terms of protection [94]. However, these methods have a tradeoff when it comes to performance, which may cause the final model to be inaccurate [48]. Furthermore, future techniques and additional information may compromise a previously low-risk model.

#### 6.1.2. Level of Trust

FL collaboration can be classified into two broad types:

  Trusted: FL consortia, whose partnership is binding and regarded as trustworthy, typically prevent a few serious reasons, like a plot to steal crucial data or damage their system. To reduce the necessity of specialized countermeasures, researchers can reexamine the fundamentals of regular cooperation studies [95].
  Nontrusted: FL systems that are used in a wide range of settings could prove difficult to develop a collaborative partnership that guarantees timely, appropriate collaboration between all parties. In some cases, users may attempt to misuse the system, undermine its performance, or obtain information from others intentionally. In order to reduce these threats, security strategies including cryptography of product proposals, effective authorization for participants, tracking of operations, asymmetric security, conducting testing, performance reliability, confidentiality of design, and protection from opponents are necessary [96].

#### 6.1.3. Data Breach

Patients' medical information is usually not shared across FL devices and hospitals. Nevertheless, the observations can indeed effectively leak personal information required for regional development, such as reversal [97] model updates, gradients [98], and opponents.

Traditional training differs from FL in that multiple parties are exposed to the training process. Therefore, monitoring alterations in a model over time, monitoring exact model upgrades, or manipulating a model can occur as a consequence of a dynamic analysis breach.

### 6.2. Data Heterogeneity

In addition, caused by things like data gathering methods, company biomedical applications, and area demographics, medical data differs in types, sizes, and attributes. In light of this, algorithms and strategies apply to FL as an important factor: many traditional techniques distribute relevant information independently and in the same way to all users (IID). Given the fact that there can be no comprehensive information distribution among institutions [56, 62], FL can be trained using non-IID data. In such conditions, techniques such as FedAvg [47] frequently fail, which violates the fundamental aim of active learning. Researchers have been studying this issue by utilizing FedProx [99] and part-sharing strategies [100]. Furthermore, heterogeneity of data may lead to a situation in which local solutions are not optimal for global solutions. It is therefore crucial that all participants agree on the definition of model training optimality before the training begins.

### 6.3. Traceability and Accountability

FL in healthcare is a system that needs to have reproducibility as it is a safety-critical application. Running multiparty computations in complex hardware, software, and networking environments is different from training with centralized data. In order to fulfill the traceability requirement, it must be possible to trace network connections, connectivity records, and configuration modifications, including hyperparameter tuning, throughout the training process. Additionally, traceability can be logged information about a model's training history, particularly to ensure training and test datasets are not overlapping. Execution integrity is fundamental to traceability and accountability in nontrusted federations [31].

Using FL, researchers cannot view pictures of designs that are generated. The individual sites still have their unique original information available, but organizations can choose to give any central viewing facility to meet necessity or indeed make it possible to explain and interpret the optimization method.

### 6.4. System Architecture

Computing resources and networks are typically better suited to large-scale FL in healthcare institutions than in consumer devices [31]. These methods make it possible to teach a greater number of products, and also the data collected with these methods can be shared more widely. As a result, FL brings both opportunities and challenges to healthcare, such as the following:

- It is crucial to examine when discussing whether safety prevention is achievable (e.g., by providing unneeded packages).
- How should encryption methods be designed to utilize computational resources effectively?
- Which methods can be used to reduce idle time and to take advantage of independent systems in order to develop suitable network configurations?

### 6.5. Client Management

Client management is an essential issue in FL, in contrast to the centralized ML architecture. Some clients may be looking for benefits without contributing; it is the responsibility of the server to decide which customers should participate in the learning process.

Client management involves helping a patient or client develop a plan that coordinates and integrates essential support services for the most optimal results and outcomes [101]. There are many components of client management. Hudon et al. summarize several descriptions of case (client) management, including those of the Case Management Society of America and the Canadian National Case Management Network, and describe six core elements that include patient identification and eligibility determination, assessment, care planning along with goal setting, plan implementation, plan monitoring, and transition and discharge [102].

### 6.6. Health Dataset Issues

The healthcare sector has the capability to deal with a wide range of data types and content, including text, images, audio, and time series, as well as blood types, heart rates, facial images, and body temperatures. The majority of FL approaches are typically examined on a single dataset with a limited number of features. However, despite the fact that both are proposed for privacy-preserving FL-based healthcare services, the work in [103] is tested on a dataset related to diabetic retinopathy, and the work in [69] is evaluated on an EHR dataset. The central server can navigate heterogeneity by using private ensemble learning [104] to navigate heterogeneous FL approaches involving multiple parties having different models. An inference strategy is presented to enable participants to use an ensemble of heterogeneous models without needing to explicitly join the data in a single place.

A summary of the key findings from the above discussion can be found in Table 6.

#### 6.6.1. Future Directions

In the future, healthcare tasks may be performed better using collaborative strategies across multiple institutions, as opposed to very large files limited to a specific health center. FL can integrate information acquired and maintained by different institutions in order to capture broader data variability and analyze patients from diverse demographics. Furthermore, FL allows the addition of multiexpert annotation and data from multiple sites acquired with different instruments and techniques. In order to achieve this collaborative goal, different agreements must be put in place, including details about scope, purpose, and technology, which may not be known at present as the field is a new one.

#### 6.6.2. FL for Healthcare 5.0

In the traditional ML model, the data used in training the models raised issues regarding security and privacy. In this context, FL has been found to enhance scalability, improve accuracy, reduce training time, improve performance, increase privacy and enhance safety, among others [105]. FL has some advantages over the traditional methods, which are as follows:

- An FL network makes the entire network scalable by enabling different devices to learn from one another.
- The creation of local models reduces latency and lowers the power consumption when compared to training a single central model.
- As a result of their use of many local models and simultaneous perspective approaches, FL models are more innovative than centrally trained models.
- FL provides predicted values in real time because you can access the datasets without a centralized server. It reduces the lag time in data access and enables you to access data without connecting to the central server. Data can be transmitted and received directly through a local server.
- Compared with standard central ML algorithms requiring datasets on a single server, FL reduces security and confidentiality concerns by managing local database objects.

#### 6.6.3. FL for Digital Twin in Healthcare

In the future, healthcare will become automated, citizens will adopt a more proactive approach to their health (empowerment, prevention), and clinical decision support will be integrated throughout practice. The combination of these measures will allow the healthcare system to be sustainable in the future, even when the population grows older.

Several applications of digital twin technology are found in the healthcare sector. In healthcare, technology is enabling advances that, once thought impossible, are becoming possible thanks to its growth and development. The health sector uses many applications that do not directly benefit the patients but have a beneficial influence on how they are treated. Therefore, these systems play a vital role in improving patient care.

Healthcare is even more dependent on simulation and actuations in real time as it can mean the difference between life and death. In addition to predictive maintenance and ongoing equipment repair, the digital twin can also assist with the diagnosis and prevention of problems. Medical digital twins can make life-saving decisions on the basis of real-time and historical data with the help of AI [106, 107].

Regulations are one step to ensuring personal data is protected, but FL is another way to build decentralized training models. The privacy and security issues associated with data analysis within a digital twin are addressed by keeping users' data localized within an FL model, which provides the desired outcomes in terms of implementing data analytics [108].

#### 6.6.4. FL and Blockchain for Healthcare

The FL-based technique would be able to help the healthcare providers because it can improve the accuracy and robustness of the AI model and that helps to make the model more generalizable so that it could be used in the real-time environment [109]. At the same time, this approach would be able to save time and cost. The most important part is that we would be able to get all the benefits from FL without leakage of information.

The software and hardware manufacturers would get a lot of benefits from the FL because collaborative learning is possible between hardware devices and software applications without information leakage and the other benefit will come in terms of validation of the AI-based model since it is combined with the hardware devices that helps in continuous improvement of AI-based models [110].

Researchers and developers get a lot of benefits from the FL-based services because a huge amount of real data would be available to them [111]. This will help the AI researchers and developers to think about different algorithm strategies that would help them to make robust models.

#### 6.6.5. FL for Collaborative Robotics in Healthcare

Collaborative robotic (Cobot) technology has been widely adopted both by healthcare professionals and by those involved in the medical device industry as a tool to improve workforce efficiency, facilitate workflow improvements, and streamline safety procedures [112].

Healthcare collaborative robots are automated systems deployed in the medical industry to perform various tasks, starting from administrative tasks, lab testing, patient care, and surgical aids. These cobots require an hour to fill the gap between the ongoing medical industry burden and staff shortage. The rising requirement to facilitate automation in the healthcare industry to reduce infection exposure to front-line workers along with increased technological advancement in inpatient care services has influenced the healthcare collaborative robots market growth.

The cobots are proved to be extremely efficient while performing the lab testing tasks. High precision, fast turnaround time, and reduced dependency on manual tasks will positively influence the lab testing applications. The other key promising federated application is patient care which includes medicine dispensing, taking swab samples, checking temperature and blood pressure, and conducting various sample tests which have made it easier for health workers to reduce their burden and utilize more time on urgent matters.

#### 6.6.6. FL and Explainable AI for Healthcare

IoT has transformed the healthcare domain by introducing the Internet of Medical Things (IoMT); however, choosing analysis for distributed IoMT environment, analysis of the enormous amount of data generated by IoMT devices in a distributed environment, and achieving security of IoMT devices is a challenging task. However, recent researches on AI-enabled remote health monitoring systems were able to monitor and prevent cyberattacks. Explainable AI is a promising modern technology for identifying the compromised data during cyberattacks in IoMT-based patient monitoring systems by enabling caregivers to fix the problems. Also, the FL-based Wearable, explainable AI frameworks will enable the user to have better communication using knowledge-based methods and also improve user acceptance and task performance [113].

#### 6.6.7. FL for Integration with 6G in Healthcare

The enormous usage of IoMT devices in our daily activities results in an explosive growth of data traffic, ML, and data-driven approaches. Moreover, the surge in the development of communication technology led to the in-novation of 6G networks, by transforming wireless communication from “connected things” to “connected intelligence,” expecting to embody advanced AI various applications promising grater-level of security and stronger privacy protections in the healthcare domain. A large number of IoMT devices with massive data in the 6G era will force individuals to deploy efficient ML and AI-based algorithms to provide high-quality services. However, implementing FL-based Edge intelligence in 6G will bring in improved performance, ultralow latency service, and enhanced privacy of the system [114].

## 7. Conclusion

FL approach is a promising method for achieving strong, precise, safe, robust, and unbiased modeling results. FL facilitates communication between various parties without the need to communicate or centralize data, thereby solving difficulties associated with critical health information. In addition, this could lead to new business opportunities and improved healthcare worldwide. In this paper, the authors addressed the health sector's opportunities and drawbacks for FL. The FL investigation is expected to continue for a further decade since not every fundamental barrier has been removed recently. Despite this, we think precision medicine will have a great deal of impact on medical care in the future.

## Figures and Tables

**Table 1:** Summary of Important surveys on FL in healthcare applications.

Ref.	Applications/use cases	Requirements/vision	Technicalchallenges	Enablingtechnologies	Researchdirections	Remarks
[30]	Low	Medium	Medium	Low	Low	Focused mainly on how FL can be applied in healthcare

[31]	Low	High	Medium	High	Low	(i) Focused mainly on FL initiatives related to digital health
						(ii) It highlights FedAvg and FedProx algorithms

[11]	Medium	Medium	High	High	High	Focused mainly on the cur- rent state of FL, including but not limited to the healthcare sector

[32]	HIGH	HIGH	MEDIUM	HIGH	HIGH	Focused mainly on advanced FL designs that would be useful for federated smart healthcare, such as (i) Federated EHRs management(ii) Federated remote health monitoring (iii) Federated medical imaging, and (iv) Federated COVID-19 detection

[33]	HIGH	MEDIUM	MEDIUM	HIGH	HIGH	Focused mainly on the systematic literature review of current research about FL in the context of EHR data for healthcare applications
This paper	HIGH	HIGH	High	High	High	A comprehensive survey of FL applications for medical image processing, FL toward privacy and security in healthcare applications, FL in IoT-based smart healthcare applications and FL for outbreak prediction, technical challenges, enabling technologies and future research directions

**Table 2:** List of abbreviations.

Abbreviation	Description
ML	Machine learning
FL	Federated learning
EHR	Electronic health records
ADMM	Alternating direction method of multipliers
DL	Deep learning
AI	Artificial intelligence
IoT	Internet of things
FTL	Federated transfer learning
DT	Digital twins
MRI	Magnetic resonance image
CFL	Clustered federated learning
HCM	Hypertrophic cardiomyopathy
HIPAA	Health insurance portability and accountability act
NN	Neural network
ADR	Adverse drug reactions
FADL	Federated autonomous deep learning
AKI	Acute kidney injury
CT	Computed tomography
IoMT	Internet of medical things

**Table 3:** FL applications for medical image processing.

Ref. No	Technologies used	Key contributions	Limitations
[56]	Differential privacy techniques	Using the BraTS dataset, assess the usefulness of practical FL methods for segmenting brain tumors	It is impossible to collect and share patient data in a centralized data lake

[58]	CFL-based collaborative learning framework	To highlight the potential of intelligent processing of clinical data at the edge, open research issues related to deploying ML at the edge for healthcare applications that re- quire further investigation	(i) Image size and quality
			(ii) Contrast and brightness level, and
			(iii) Positioning of subjects

[59]	Skin imaging technology	The proposed model contains two core contributions:	Health practitioners usually apply manual or computer vision-based tools for skin tumor diagnosis, which may cause misinterpretation of the disease and lead to a longer analysis time
		(i) The model was deployed on the cloud server, and	
		(ii) Its deployment on the edges majorly contributes toward adaptability by continuously updating	

[60]	FL techniques	The performance of FL may be enhanced with more images or data augmentation	Comparisons of FL with unequal data distribution, data augmentation, and one-shot learning are required to explore the implications of data imbalance

[62]	3D-convolutional neural network technique	FL study on cardiovascular magnetic resonance diagnosis and demonstrate that FL performance is comparable to central database server	Patient privacy

**Table 4:** FL in IoT-based smart Healthcare applications.

Ref. No	Technologies used	Key contributions	Limitations
[61]	Federated semantic segmentation models	In this study, federated semantic segmentation models performed on multimodal brain scans are similar to models trained for data sharing	Data acquisition is a major challenge

[63]	DL techniques	The objective of this review is to present an overview of current research on applying DL to clinical tasks derived from EHR data, in which we examine the variety of DL techniques and frameworks applied to various types of clinical tasks	(i) Model interpretability
			(ii) Data heterogeneity, and
			(iii) Lack of universal benchmarks

[64]	A descriptive and inferential statistical analysis	The purpose of this survey was to assess electronic communication and awareness of HIPAA privacy and security rules, especially in the context of text messaging	(i) First, there was a low response rate, raising concern for nonresponse bias
			(ii) Second, survey results may be skewed by cognitive biases

[67]	Federated- autonomous deep learning (FADL) method	This study finds that FADL exceeds traditional federal methods of learning and that balancing global to local formation is an important feature of distributed techniques, especially in the field of healthcare	Accessing data is complex and slow due to: (i) Security
			(ii) Privacy
			(iii) Regulatory and
			(iv) Operational issues

[69]	FL framework	This study reveals that while differential privacy in a federal system is commonly adopted, it can lead to considerable losses in model performance in healthcare applications	(i) Distributed data silos
			(ii) Privacy issues

[70]	An FL framework can develop global ADR prediction models, based on local health data held at different locations	In this study, we focused on algorithms conducive to distributed solutions, including gradient descent, as a method supported by FL	Frameworks for predicting adverse drug reactions (ADR) using centralized learning

[78]	Blockchain and AI	In this study, we have provided a comprehensive coronavirus (COVID-19) investigation utilizing blockchain and AI	The challenges are analyzed in this article from four different perspectives: (i) Regulatory considerations
			(ii) Maintaining people's privacy
			(iii) The security of blockchain and AI ecosystems, and
			(iv) A lack of unified databases

**Table 5:** FL for COVID outbreak prediction.

Ref.No	Technologies used	Key contributions	Limitations
[19]	Blockchain-based FL framework	Training a global, more accurate ML model on hospital data can assist in detecting COVID-19 cases during lung screenings	It is challenging to share data securely (without compromising the privacy of users) and to train global models for -detecting positive cases

[20]	UCADI framework	A decentralized model, the unified CT-COVID AI diagnostic initiative, distributes and performs the AI model at each participating institution independently without sharing personal data	(i) Data deficiency
			(ii) Data isolation
			(iii) Data heterogeneity

[38]	AI and big data	The coronavirus disease COVID-19 is being controlled with the use of AI and big data	Privacy and security issues due to insufficient standard datasets

[39]	AI	In the fight against COVID-19, AI can contribute in six ways: (i) Early warnings and alerts	(i) Too much, and (ii) Too little (iii) Data
		(ii) Tracking and prediction	
		(iii) Data dashboards	
		(iv) Diagnosis and prognosis	
		(v) Treatments and cures, and	
		(v) Social control	

[78]	Blockchain and AI	The coronavirus (COVID-19) epidemic can be combated using AI and blockchain technology	The lack of unified databases is a concern for protecting the privacy and the security of blockchain

[90]	AI-related technologies	A comparison of FL to training without an FL framework was conducted using four different models: (i) MobileNet	FL presents a number of statistical and system challenges when distributed device networks are used to train machine models
		(ii) ResNet18	
		(iii) MoblieNet, and	
		(iv) COVID-net	

[91]	A novel collaborative city DT framework	FL combined with city DTs alleviates the data sparsity challenge, facilitates collaboration, and provides privacy protection by design	Collaborative training problems, such as: (i) Disaster surveillance and
			(ii) Prediction

**Table 6:** Challenges and possible solutions.

Challenges	Reason for challenges	Possible solutions
Confidentiality and safety	The fundamental issue with standard ML/DL models is that data from personal devices, sensors, and wearables from patients must be uploaded to a cloud server in order to train the data using the ML/DL models	In FL, instead of transferring data to the central servers, the ML model itself is deployed to each device to be trained on the data

Data heterogeneity	Healthcare data is heterogeneous for a number of reasons:	FL addresses the problem of heterogeneity by utilizing FedProx
	(i) Differences among patient populations	
	(ii) Environments	
	(iii) Practices, and	
	(iv)Treatment protocols	

Traceability and accountability	In FL, one of the biggest challenges is ensuring that the global ML model can be traced throughout the underlying ML process	(i) Traceability should be ensured during the training process to permit tracking of system events
		(ii) Data access history and training configuration changes, such as hyperparameter tuning

System architecture	Using a client device that provides training and communication to the model can be difficult, which can lead to low-quality models	Healthcare institutions have usually better computing resources and high-speed networks compared to consumers, so they can run FLs at scale

Client management	Client management is an essential issue in FL, in contrast to the centralized ML architecture	Client management involves helping a patient or client develop a plan that coordinates and integrates essential support services for the most optimal results and outcomes

Health dataset issues	The majority of FL approaches are typically examined on a single dataset with a limited number of features	An inference strategy is presented to enable participants to use an ensemble of heterogeneous models without needing to explicitly join the data in a single place

## Data Availability

No data were used to support the findings of the study.
